# Nonhuman gamblers: lessons from rodents, primates, and robots

**DOI:** 10.3389/fnbeh.2014.00033

**Published:** 2014-02-11

**Authors:** Fabio Paglieri, Elsa Addessi, Francesca De Petrillo, Giovanni Laviola, Marco Mirolli, Domenico Parisi, Giancarlo Petrosino, Marialba Ventricelli, Francesca Zoratto, Walter Adriani

**Affiliations:** ^1^Goal-Oriented Agents Lab (GOAL), Istituto di Scienze e Tecnologie della Cognizione, Consiglio Nazionale delle Ricerche (ISTC-CNR)Rome, Italy; ^2^Department of Environmental Biology, University of Rome “La Sapienza”Rome, Italy; ^3^Department of Cell Biology and Neurosciences, Istituto Superiore di SanitàRome, Italy; ^4^Bambino Gesù Children’s Hospital IRCCSRome, Italy

**Keywords:** pathological gambling, risk sensitivity, uncertain reward, animal models, nonhuman primates, neurocomputational models, evolutionary models

## Abstract

The search for neuronal and psychological underpinnings of pathological gambling in humans would benefit from investigating related phenomena also outside of our species. In this paper, we present a survey of studies in three widely different populations of agents, namely rodents, non-human primates, and robots. Each of these populations offers valuable and complementary insights on the topic, as the literature demonstrates. In addition, we highlight the deep and complex connections between relevant results across these different areas of research (i.e., cognitive and computational neuroscience, neuroethology, cognitive primatology, neuropsychiatry, evolutionary robotics), to make the case for a greater degree of methodological integration in future studies on pathological gambling.

## Introduction

Gambling can be defined as betting money, or other equivalent goods, upon the future outcome of an event which presents a high degree of uncertainty, with a view to winning a prize. Winning is mainly (or exclusively) due to chance and not much (or not at all) to individual abilities. While betting may represent a recreational activity for the majority of people, it may become a serious behavioral disorder for others (Petry et al., 2005). The rapid worldwide growth of legalised gaming opportunities (Wilber and Potenza, 2006; McCormack et al., 2012; Donati et al., 2013), including the increasing possibility of online gambling through the Internet, has raised concerns over the impact of exaggerated gambling and its detrimental consequences on public health (Shaffer and Korn, 2002; Carragher and McWilliams, 2011). Thus, due to the increasing number of affected people, pathological gambling represents a growing concern for society.

In fact, this behavior is clinically characterized as a pathology: in DSM-IV-TR (American Psychiatric Association, 2000), it was described as a persistent, recurrent and maladaptive behavior, which disrupts personal, family, professional or vocational pursuits (Potenza, 2001). The personal and social consequences of this disorder often include job loss, family problems and divorce, financial and legal problems, and criminal behavior (Lowengrub et al., 2006). Pathological gambling affects 0.2–5.3% of adults in western socities (Bastiani et al., 2013) and is highly comorbid with a range of other psychiatric disorders such as attention-deficit/hyperactivity disorder (ADHD; and other impulse-control disorders, obsessive-compulsive disorders; Hollander et al., 2005) and with substance abuse (Petry et al., 2005; Hodgins et al., 2011). Some pathological features of gambling are similar to those of drug addiction, such as the need to gamble increasing amounts of money (escalation) in order to achieve the desired excitement or “rush” (tolerance), the irritability that accompanies the abstention from the activity (withdrawal), the failure of attempts to control or stop the behavior (loss of control). Notably, whilst pathological gambling has been classified until recently (in DSM-III and DSM-IV) among the “Impulse-Control Disorders Not Elsewhere Classified”, it has been turned into a “no substance addiction” in DSM-V (American Psychiatric Association, 2013), that is a “behavioral addiction”. Pathological gambling is also associated with increased suicidal ideation and attempts compared to the general population: approximately one out of five pathological gamblers attempts suicide (Volberg, 2002). Such rates among pathological gamblers are higher than for any other addictive disorder. Thus, gambling represents a public concern being both a social and a psychiatric issue.

Far from being an adult concern, gambling is becoming a serious behavioral problem also among adolescents (Cunningham-Williams and Cottler, 2001; Dickson et al., 2002), whose involvement has increased substantially over the past 20 years (Huang and Boyer, 2007). Epidemiological studies show that the prevalence of pathological gambling is 2–4 times higher among adolescents than among adults, with 3.5–8.0% of adolescents meeting the criteria for such pathology (Felsher et al., 2004; Ellenbogen et al., 2007; Hodgins et al., 2011; Caillon et al., 2012). Adolescence and young adulthood may be periods of especially heightened vulnerability for the development of gambling disorders, which are therefore receiving increasing attention by clinicians and preclinical researchers (Jazaeri and Habil, 2012; Zoratto et al., 2013).

The etiology of pathological gambling is multi-factorial; both genetic (e.g., a polymorphism in the serotonin transporter gene; Ibanez et al., 2003) and socio-environmental (e.g., Donati et al., 2013; Potenza, 2013) risk-factors have been identified. Moreover, cognitive models of gambling argue that irrational beliefs and erroneous perceptions may play a key role (Reid, 1986; Clark, 2010). Indeed, some authors argue that expectancies of winning, illusions of control, and subsequent entrapment do contribute to the development and the maintenance of gambling patterns (Joukhador et al., 2003). Psycho-genetic studies have revealed that, among genes involved in altered serotonergic and dopaminergic neurotransmission, the most significant for pathological gambling are serotonin transporter (SERT; Ibanez et al., 2003; Reuter et al., 2005) and dopamine transporter (DAT; Comings et al., 2001).

Methods for treating pathological gambling include various counselling-based approaches and pharmacological therapy, although there are no drugs which have been officially approved for the specific treatment of pathological gambling by the U.S. Food and Drug Administration (FDA). Therefore, in pathological gamblers, drugs are mainly prescribed for the treatment of the comorbid conditions and not for the pathology itself (Hollander et al., 2005). Pathological gamblers respond well to treatment with selective serotonin reuptake inhibitors (SSRIs, particularly paroxetine; Kim et al., 2002), mood stabilizers, and opioid antagonists (such as nalmefene), commonly used in the treatment of alcoholism (see for a review Lowengrub et al., 2006).

In view of the growing incidence of pathological gambling, its severe mental and social consequences, and the still preliminary nature of its treatment, it is urgent to mobilize various approaches and methods to further deepen our understanding of the neuronal and psychological underpinnings of this condition. Indeed, the present Research Topic constitutes an important and timely initiative towards that end. The contribution we offer in this review concerns how evidence obtained on nonhuman subjects is crucial to investigate pathological gambling in humans. In particular, we make the case for studying three widely different populations of agents: rodents (Section Rodents as an Animal Model of Gambling Behavior), nonhuman primates (Section Risky Choices in Nonhuman Primates: Implications for Human Pathological Gambling), and robots (Section Risk Attitudes, Environmental Uncertainty and Addictive Behavior: Perspectives From Computational Neuroscience and Evolutionary Robotics). While each of these populations offer valuable insights on the topic, their true worth is revealed only by looking at how they relate to each other. Hence we will review the literature across all these areas of research (i.e., cognitive and computational neuroscience, neuroethology, cognitive primatology, neuropsychiatry, evolutionary robotics), with the aim of suggesting the need for greater methodological integration in future studies on laboratory modeling of pathological gambling.

## Rodents as an animal model of gambling behavior

In the field of behavioral neuroscience, animal models enable the investigation of brain-behavior relations under controlled conditions (e.g., standardized housing and testing), with the aim of gaining insight into normal and abnormal human behavior and its underlying neural, psychobiological and neuro-endocrinological processes (van der Staay, 2006). In particular, they are particularly suitable for the dissection of precise mechanisms involved in decision-making processes, for the analysis of inter-individual differences with a tight control of environmental and genetic conditions, and for follow-up studies (de Visser et al., 2011). As we shall see in what follows, these considerations do apply also to the study of gambling behavior, and especially to the use of rodents (mostly rats) as an animal model for risk proneness (e.g., Adriani et al., 2009, 2010).

### Assessment of gambling proneness: clinical and preclinical approaches

In humans, Probability Discounting can be studied by means of either questionnaires or operant paradigms. The “South Oaks Gambling Screen” (for adults Lesieur and Blume, 1987; for adolescents Wiebe et al., 2000), the “Gambling Attitudes and Beliefs Survey” (Strong et al., 2004) and the “Canadian Problem Gambling Index” (Young and Wohl, 2011) are some examples of personality tests and reports, widely used in the framework of clinical psychology and experimental research. In these protocols, gamblers are characterized with scores that represent their averaged behavior over periods of weeks, months or years whilst the time spans that most naturally correspond to the expression of gambling behavior are those of seconds, minutes or hours. The main limitation of these traditional methods regards therefore the lack of an appropriate temporal dimension (van den Bos et al., 2013). By contrast, controlled experimental or clinical paradigms such as the “Iowa Gambling Task” (IGT; Bechara et al., 1994), the “Balloon Analogue Risk Task” (Lejuez et al., 2002) and the “Probability Discounting Task” (e.g., Scheres et al., 2006; Shead and Hodgins, 2009) allow to overcome the above mentioned limitation regarding the temporal dimension. However, as extensively discussed in van den Bos et al. (2013), they are characterized by a second limitation, i.e., the lack of appropriate context due to the artificial conditions of a laboratory environment. It should also be noted that these paradigms can be performed with either real rewards over limited time intervals (e.g., minutes, hours) or with questions about hypothetical ones (e.g., huge amounts of money) over months or years.

Due to the complexity of human studies, preclinical investigations in laboratory animal models are necessary for a deeper understanding of pathological gambling. Specifically, it is relevant to exploit preclinical models for (i) the symptoms; (ii) their neurobiological determinants; and (iii) their possible modulation by pharmacological manipulation. Specifically, these studies are crucial as they allow the dissection of processes and factors associated with normal and pathological gambling in a controlled way (de Visser et al., 2011; Winstanley et al., 2011; Koot et al., 2012). Furthermore, animal models have added value from a translational perspective because it is possible to use approaches that are virtually impossible with humans, as in the case of *in vivo* transgenic approaches that allow to directly reach and modulate expression of target genes in relevant brain areas (Adriani et al., 2010).

Many operant paradigms have been developed to study tolerance to uncertainty and/or gambling proneness in animal models (Mobini et al., 2000; Cardinal and Howes, 2005; Adriani et al., 2006; Wilhelm and Mitchell, 2008; Winstanley et al., 2011). Specifically, by exploiting uncertainty of reward delivery, these tasks allow to probe individual (in)tolerance to frustration, linked to missing an anticipated reward (i.e., the “loss”). The “IGT” involves the choice between a low probability of a large reward vs. a high probability of a small food reward (van den Bos et al., 2006). The “Probabilistic-Delivery Task” (PDT; which belongs to the broader category of Probability Discounting) is based on a choice between either a certain, small amount of food reward or larger amounts delivered (or not) depending on a given (and progressively decreasing) probability (Adriani and Laviola, 2006; Adriani et al., 2006). The “Risky Decision-Making Task” (RDT) implies the choice between a small, “safe” food reward or a larger food reward associated with the risk of punishment (e.g., footshock; Simon et al., 2009). The “rodent Slot Machine Task” (rSMT) allows to evaluate if the experimental subject discriminates a complete signal (e.g., three lights turned on, indicative of win) from a nearly complete one (e.g., two lights out of three, indicative of loss): by means of this task, it has been recently demonstrated that rats are susceptible to putative-win signals in non-winning trials (Winstanley et al., 2011; Cocker et al., 2013). Such a phenomenon might resemble the so-called “near-miss effect”, one of the cognitive distortion regarding gambling outcomes that is thought to confer vulnerability to pathological gambling (Reid, 1986; Clark, 2010; see also Section Normative (Algorithmic) Models).

Notably, the “IGT” and the “Probability Discounting Task” are widely used in experimental or clinical research on humans. Obviously, when performed on animals, these paradigms involve real, ethologically relevant rewards over limited time intervals. Symbolic reward (as money in humans) or time intervals longer than few hours cannot be used. Moreover, to be effective, the contrast between alternative rewards (e.g., small vs. large one) can not be as marked as it would be desired to mimic 1000-fold prizes as in humans. In these tasks, in which a moderate food restriction is usually applied to increase subjects’ motivation to work for food delivery, the rewards’ magnitude shall be accurately calibrated in order to (i) allow animals to eat enough food; (ii) prevent them from being fully satiated; and (iii) enable them to discriminate between rewards. The first aspect is especially relevant in “closed” (compared to “open”) economies, in which subjects have to obtain all their daily meal from the operant panels and no extra food is given at the end of each experimental session (Timberlake and Peden, 1987; Zoratto et al., 2012). The second one is necessary to avoid a potential recovery from the consequences of the food loss (occurring because of the probabilistic delivery). The last one can be crucial for the establishment of basal preference in developing rats (Zoratto et al., 2013). We have recently shown that high contrast between rewards (one pellet vs. five pellets instead of two pellets vs. six pellets) and high probability initially associated, during training, with the large reward (66% instead of 50%) are essential to shorten the overall testing period: namely, much less sessions are required for the development of baseline large-reward preference (which is otherwise slow in young animals). This is of paramount importance to overcome the developmental constraint associated with the short duration of the adolescent phase (Laviola et al., 2003).

These operant-behavior tasks imply a series of discrete decisions between two reward alternatives (Adriani et al., 2012a). In terms of automatization, the experimental apparatus requires two alternative *operanda* (e.g., levers or nose-poking holes, where the animal can express its choice), and computer-controlled delivery of reinforcers (e.g., food or liquids) that differ in size and actual probability of delivery (uncertainty). Other important features of the task are inherent to the trial/session schedule. For instance, the total number of choice opportunities (i.e., trials) given to the subject may be fixed (i.e., the session ends after the last trial) and independent of total time needed to complete the task. Alternatively, the total duration of the experimental session may be fixed (minutes, hours) and thus independent of the total number of trials actually completed within such time-window (Koot et al., 2012).

The protocols reviewed above probe animals for the balance between “innate, sub-cortical” drives and “evolved, cortical” processes (Adriani and Laviola, 2009). In other words, these operant tasks allow to evaluate a cognitive ability, i.e., to inhibit sub-cortical drives and to express a more controlled response. Self-control is known to require intact serotonergic function (Wogar et al., 1993; Harrison et al., 1997; Puumala and Sirvio, 1998; Dalley et al., 2002), especially within the prefrontal cortex (McClure et al., 2004; Ridderinkhof et al., 2004) and its cortico-striatal projections (Cardinal et al., 2004; Christakou et al., 2004).

### The probabilistic-delivery task (PDT)

The “PDT” (Mobini et al., 2000; Adriani and Laviola, 2006) involves a larger but probabilistic reinforcer which is randomly withheld by the feeding device, and delivered only occasionally so that experimental subjects face a “loss”. The progressively accumulating “losses” over time clearly have consequences for the sake of long-term payoff. Such a task also provides information reflecting the ability to cope with non-regularly delivered, randomly missing reinforcement. We have shown recently that laboratory rodents are not only tolerant to this random delivery, but are also sub-optimally attracted by this probabilistic uncertainty (Adriani and Laviola, 2006, 2009). Indeed, if the very frequent food-delivery omission is masked by the same cue (e.g., a light flash) normally accompanying occasional food delivery, this cue may turn out to act as a secondary reinforcer. As such, like in second-order schedules, this conditioned stimulus may sustain continued responding for the large/uncertain reward, even though this implies a decreased overall foraging in the long term. Gambling proneness may thus be sustained by the cue-induced secondary reward, which renovates in the subject the expectation for an eventual delivery of binge reward (Adriani and Laviola, 2006, 2009). Translated to human subjects, this would suggest that it is the thrill—associated with whatever physical stimuli accompanies both successful and unsuccessful gambling experiences—that sustain a motivation to gamble, in spite of abysmal odds and past (mostly negative) experience: looking at the ball madly spinning on the roulette and waiting for the crucial card to be turned, with a mix of hope for success and fear of loss, become rewarding in themselves, and it is in view of these (certain) rewards that people start enjoying gambling activities. Until the individual can keep under control the desire, these activities have nothing wrong in themselves. However, in vulnerable individuals, eventually a loss of control over these activities may intervene: pathological gamblers keep on gambling as this compulsive “urge” becomes a strong habit, not differently from other kinds of addictions (van den Bos et al., 2013).

#### Methodological remarks on the probabilistic-delivery task (PDT)

A theoretical framework has been recently formulated to interpret the performance of laboratory rats in this kind of two-choice tasks (Adriani and Laviola, 2006). Specifically, a landmark in the PDT protocol is the “indifference” point: i.e., the specific level of uncertainty at which the animals can choose either option freely with no effect on the overall economic convenience. As an example, if the ratio between large and small reward size is five-fold then the indifference point is at “*p*” = 20%. Therefore, once the “indifference” point is established, the range of “*p*” values providing worthy information is easily recognized at “*p*” values beyond the indifference point (i.e., 20% > “*p*” > 0%), when economical benefit (i.e., maximization of payoff) is attained unequivocally by choosing repeatedly the small-reward option. Thus, to maximize the payoff, subjects should be flexible enough to abandon their innate large-reward preference. As optimal performance in terms of benefit takes the form of a choice-shift towards small reward, this requires a self-control effort in order to overcome the “innate drive” that justifies its attractiveness (Adriani et al., 2006). By contrast, a sustained preference for large reward denotes “temptation by risk”.

In this kind of two-choice tasks, details of the schedule can be calibrated appropriately (Adriani and Laviola, 2009), so that one alternative option leads to “optimal” benefit (i.e., the raw convenience in terms of quantitative foraging or any other measurable revenue), while the other alternative provides an “affective” benefit, with a more emotional outcome (i.e., better feeling and/or avoidance of adverse mood). In brief, to run a protocol providing useful information, any “inner drive” of interest (e.g., gambling proneness) shall push animals into a choice that necessarily leads to a sub-optimal outcome. Self-control is then defined as the ability to effect an optimal response (Stephens and Anderson, 2001) by directing choices onto the opposite *operandum* (nose-poking hole or lever to press). The protocol must never load both instances (i.e., the inner drive and the optimal payoff) on the same *operandum* because it would be impossible to discriminate whether any preference for that *operandum* is due to payoff-detecting processes (“economical efficiency”) or to the “inner drive” itself.

#### Probabilistic-delivery task (PDT) at very low probability levels

Many factors can act together to push animals towards a suboptimal preference for a large reward, even though this is delivered quite rarely. One factor is insensitivity to risk, whereby the subjects are unable (i) to figure the uncertainty in the outcome (usually, they should anticipate the notion that reward is not for sure, which acts as a source of aversion immediately before choice) or (ii) to perceive the punishment of “losses” (represented by the occurrence of a randomly and frequently omitted delivery of reward).

Another factor is habit-induced rigidity, under which the subject seems to behave according to a well consolidated strategy. Such form of inflexibility may be due to a failure of negative reinforcement, namely to a lack of adaptation and feedback-reaction to the aversion (for an anticipated “unsure” prize) and/or to the punishment (due to an actually “omitted” prize) just described.

A third factor is temptation to gamble, whereby the motivational impact of the reward magnitude (“bingeing”) seems to monopolize the subject’s attention over any other reward feature. It is also possible that risk of punishment under conditions of uncertainty becomes attractive as a secondary conditioned feature, and this because the “binge” reward (eventually delivered) may well be generating an overwhelming peak of positive reinforcement. The latter could extend a secondary rewarding property to all cues and surrounding stimuli that predict uncertain features. Whatever of these factors is prevalent in the PDT and in similar tasks, the sub-optimal preference for big, rarefied reward is taken as an index of “gambling proneness” (namely, the innate attraction for a “rare but binge” event).

### “Risk of losing” vs. “failing to win”

A crucial component of human gambling is the “risk of losing”, that is, “the resources staked on a favorable outcome are lost when a wager is unsuccessful” (Zeeb et al., 2009). This is distinct from “failing to win”, that is, the absence of any additional gain, causing a “frustration” but only compared to one’s expectation.

Most paradigms of risky decision-making (Mobini et al., 2000; Cardinal and Howes, 2005; Adriani and Laviola, 2006; van den Bos et al., 2006) deal exclusively with “failing to win”: i.e., complete omission of reward delivery, or delivery of an unpalatable reward. Thus, there is frustration of an expectation but no risk of “negative payoff”, i.e., of finishing the session at a disadvantage compared with the start. In other words, every case of unsuccess is an “unlucky event” but not necessarily a “risk”. Therefore, while the attraction for uncertain reward may resemble the features of a “gambling proneness”, it is not necessarily fitting with the construct of “risk proneness” (on this point, see Anselme, 2012). Therefore, it should be noted that “uncertainty” and “risk” are not synonymous:^1^ indeed, the PDT and similar tasks do offer stochastic “unsuccess” which is even a “punishment”, but not necessarily a “risk” which would need a construct implying a potential for overtly adverse consequences (e.g., footshock).

Recently, however, choice behavior has been also studied in a setting where a greater reward was associated with the probability of an overtly adverse event (i.e., the “risk”), represented by a foot shock (Simon et al., 2009). This can represent a promising methodological refinements of paradigms tailored for gambling proneness, although its ethical implications (especially when dealing with non-human primates) should be carefully evaluated.

Another attempt to deal with this issue is represented by the “Rat gambling task” (rGT; Zeeb et al., 2009). In this task, subjects have a limited amount of time to maximize the number of pellets earned, and loss is signaled by punishing timeouts during which reward cannot be obtained. On each trial, animals can choose from four options, each associated with a different number of sugar pellets; each subject then receives either the associated reward or a punishing timeout. Larger reward options are associated with a higher chance of longer timeouts, resulting in less reward earned overall per session. To maximize their earnings, rats must learn to avoid these risky options.

### The ecological validity of animal models of human (pathological) gambling

Classically, the performance of laboratory animals on tasks tailored for gambling proneness is investigated by placing the animals (in most cases laboratory rodents, primarily rats) individually in operant chambers for a short daily session (Evenden and Ryan, 1996, 1999; Mobini et al., 2000, 2002; Adriani et al., 2009). Thus, differences across laboratories in working environments and in human interventions (e.g., handling and transport to a novel testing room) may compromise the reliability and reproducibility of behavioral data (Crabbe et al., 1999; Wahlsten et al., 2003).

Therefore, for the ecological validity of animal models of human (pathological) gambling, it is critical to address some crucial issues (van den Bos et al., 2013). Firstly, confounding factors such as stress due to handling, facing a new environment and social isolation should be avoided (e.g., de Visser et al., 2006; Spruijt and de Visser, 2006; Koot et al., 2009, 2012; Zoratto et al., 2013). Secondly, the level of tasks’ automation should be increased, since the involvement of the experimenter during testing procedures (and for scoring behavior) may be difficult to standardize: indeed, results may often strongly vary between laboratories (Crabbe et al., 1999; Chesler et al., 2002). Thirdly, tasks incorporating a social component should be used, to assess the impact of social factors on gambling proneness. It is well known, indeed, that the social environment in humans may have an undeniable effect on the development and maintenance of pathological gambling. Finally, innovative tasks should be developed that allow the investigation of normal time-budget (and its potential disruptions) devoted to social interaction, foraging, and other activities. This aspect, which is yet unexplored in animal models, would be highly relevant. The goal is to identify altered time budget possibly analogous to the disruption of personal, professional or financial life, widely reported in human pathological gamblers (DSM-IV-TR, American Psychiatric Association, 2000; Potenza, 2001).

To address the issues mentioned above, different automated social home-cage systems have recently been developed for permanent monitoring of subjects’ operant-choices and spontaneous (social and non-social) behavior (e.g., Adriani et al., 2012b). For instance, the Home-Cage Operant Panels (HOPs, PRS Italia) are new low-cost computer-controlled operant panels (Koot et al., 2009), which can be placed inside the home-cage, enabling rodents to operate it 24 h/day. Operant-choice tasks are particularly interesting to be run during adolescence (Adriani and Laviola, 2003; Adriani et al., 2004), but social deprivation during this ontogenetic period may produce changes in reward sensitivity (Van den Berg et al., 1999), as well as psychotic-like symptoms (Leussis and Andersen, 2008). To solve this problem, Zoratto et al. (2013) recently developed a considerable methodological improvement that allow testing adolescent rats in the home-cage with a task tailored for gambling proneness, while socially living and within the limited span of this developmental phase.

## Risky choices in nonhuman primates: implications for human pathological gambling

Laboratory studies in nonhuman primates can inform the research on human pathological gambling in at least four three ways. First, the behavioral tasks employed in laboratory rodents (see The Probabilistic-Delivery Task (PDT)) may be implemented in non-human primates for studying the psychobiological bases and evolutionary roots of human gambling behavior. Second, the comparison of risk preferences between phylogenetically closely related nonhuman primate species with different ecologies can shed light on the selective pressures that shaped decision-making under risk in the course of the evolution. Third, the study of how nonhuman primates make decisions under risk may provide important information on the contextual and social factors determining the occurrence of similar risky choices in humans. Fourth, since nonhuman primates are our closest relatives, but are not constrained by the socio-cultural system of beliefs and attitudes that characterizes humans, their study may allow to assess whether biases in the making of decisions under risk emerged before the human lineage diverged from the other primates, or whether they are a more recent—and possibly culturally determined—acquisition.

As noted above (see The Ecological Validity of Animal Models of Human (Pathological) Gambling), in studies with nonhuman primates, the term “risk” is typically understood as the frustration of a positive expectation (failure to receive a reward), rather than as the occurrence of a negative event (a loss of valuable resources, or the infliction of physical damage). This happens since the second type of “risk” cannot be implemented in nonhuman primate experiments, mostly due to ethical considerations. However, it is clear that nonhuman primates are exposed, in their own environment, also to “true” risks of the second type (e.g., predation). Note that, in humans, the risks involved in pathological gambling include the loss of job, family, social reputation; in a laboratory model, the appropriate meaning of “risk” should encompass therefore the possibility of overtly adverse outcomes as consequence of “high stakes”. In any case, a comparative approach has much to offer to our understanding of human attitudes towards such “high stakes risks”, once appropriate methodologies for studying them will be developed.

### The probabilistic-delivery task (PDT) in the common marmoset

The behavioral tasks mentioned in Section Rodents as an Animal Model of Gambling Behavior, used to focus on particular gambling-related aspects, are classically performed in laboratory rodents, primarily rats. However, the implementation of these tasks in species other than rats (that is, non-human primates) may be relevant for studying the psychobiological bases and evolutionary roots of human gambling behavior. Moreover, very little is known about the possibility to run such tasks by means of automated operant panels. This possibility is especially relevant in sight of increasing the ecological validity of these models (see above). The HOPs, originally developed for rodents, have been recently adapted to small non-human primates like the common marmoset (*Callithrix jacchus*; Adriani et al., 2013). In such a recent experiment, whereby the *operandum* was adapted for example into hand-poking holes, we showed that HOPs can be reliably exploited to model operant-choice behavior in a delayed-reward setting. The aim of future studies will be to evaluate marmosets as possible models for gambling behavior, using a PDT and drawing a comparison with rats.

### The “ecological rationality” of risk preferences

According to normative economic models, mainly formulated in mathematical terms, rational decision makers should be indifference when choosing between a safe option and a risky option leading on average to the same payoff (e.g., von Neumann and Morgenstern, 1947). In practical terms, this means that a rational decision maker has no reason to prefer either option when offered choice between e.g., a certain, small reward vs. an uncertain, larger one whose size is five-fold and whose probability of delivery is at “*p*” = 20% (i.e., at the indifference point). However, both human and nonhuman animals are not similar to such “rational” entity, as their instinct will guide their choice towards some kind of a preference: they are generally risk-averse for gains (e.g., Kahneman and Tversky, 1979; Kacelnik and Bateson, 1996), with the notable exception of nonhuman primates, for which the picture is more complicated (Stevens, 2010). To explain this pattern of behavior, it has been proposed that risk-related preferences could reflect the environments in which species evolved and, in particular, their feeding ecology (Heilbronner et al., 2008), leading to “ecologically rational” decisions (Gigerenzer and Todd, 1999). In order to test the above ecological hypothesis, risk preferences were compared in phylogenetically closely related primate species employing two main paradigms.

In the most simple paradigm, the subject is given a series of choices between two options: the “safe” option yields a reward that is constant in amount, whereas the “risky” option yields a reward that varies probabilistically around the mean, with the two options leading on average to the same payoff. Individuals’ attitude towards risk is inferred on the basis of their preference for the safe option (indicating risk aversion), for the risky option (indicating risk seeking) or for neither option (indicating risk neutrality) (Kacelnik and Bateson, 1996, 1997). Bonobos (*Pan paniscus*) and chimpanzees (*Pan troglodytes*), two closely related species that evolved behavioral differences possibly as a result of their different ecologies (Wrangham and Pilbeam, 2001), received an experimental schedule whereby they were offered choices between two different upside-down bowls, covering the safe option (always four food items) and the risky option (either one or seven food items with equal probability; Heilbronner et al., 2008). The two species differed markedly in their risk preferences: chimpanzees were risk-seeking, whereas bonobos were risk-averse. Their feeding ecology offers a plausible explanation for this difference: bonobos feed mainly on terrestrial herbaceous vegetation, an abundant and reliable food source, whereas chimpanzees feed primarily on fruit, a more variable food source (Wrangham and Peterson, 1996). Thus, since chimpanzees often rely on more unpredictable food sources than bonobos, this evolutive force may have shaped their behavioral regulations so that to render them tolerant to, if not attracted from, a reward uncertainty. As such, an ecological feature may have led them to be more risk-seeking than their sister species (Heilbronner et al., 2008; Stevens, 2010).

A methodologically similar study conducted on individuals belonging to different lemur species (*Lemur catta*, *Eulemur mongoz*, *Varecia rubra*) showed that, as bonobos, lemurs were clearly risk-averse (MacLean et al., 2012). Subjects were required to choose between two images on a touch-screen, associated to a safe option and to a risky option, respectively. The safe option always led to one food item, whereas the payoff of the risky option varied across two experiments. In a first experiment, the risky option corresponded either to two food items or to zero food items with equal probability (leading to an average payoff of one food item, as the risky option). In a second experiment, the payoff of the risky option was gradually increased across trials up to 7.5 times the safe option. In the first experiment, lemurs strongly preferred the safe option; in the second experiment, half of the subjects switched to risk seeking only when the potential payoff of the uncertain option was at least five times higher than that of the safe option. These results are somewhat puzzling if compared to the findings obtained by Heilbronner et al. (2008) in chimpanzees. However, it can be hypothesized that animals living in a relatively productive environment compared to lemurs, like chimpanzees, can exploit also risky resources, and thus evolve a risk-seeking attitude, without incurring in the danger of starvation. In contrast, for animals living in very harsh environments, like lemurs (that have also evolved several anatomical and behavioral traits as adaptations to their unpredictable habitats; Wright, 1999), risk proneness is not advantageous in the long term and is better to rely on low-quality, yet stable resources (Caraco, 1981; McNamara, 1996).

In a more complex paradigm, Haun et al. (2011) investigated whether, when choosing between a safe and a risky option, the four nonhuman great ape species (*Pan paniscus*, *Pan troglodytes*, *Gorilla gorilla*, and *Pongo abelii*) make decisions based on the expected value, defined as the probability of receiving the reward multiplied by the amount of the reward. In each trial, subjects choose between a safe option, consisting in a small food item hidden under a yellow cover positioned to the right of the subject, and a risky option, consisting in a large food item put in one of four brown bowls placed in a row in front of the subject and hidden under a blue cover. The probability of receiving the reward was manipulated by increasing the number of blue cups covering the four brown bowls (varying from *P* = 100%, when one blue cup covered the brown bowl containing the risky option, to *P* = 25%, when four blue cups covered all the brown bowls), whereas the relative value of the risky option was increased by decreasing the size of the small food item. Overall, apes preferred the risky option, although their preferences were influenced by the expected value. In fact, subjects chose the safe option more often when (i) the safe reward increased in size compared to the risky reward, and (ii) the probability to receive the risky reward decreased. As for species differences, chimpanzees were more risk-seeking than bonobos (as in Heilbronner et al., 2008) also when tested in this more complex paradigm, and orang-utans, whose feeding ecology is somewhat similar to that of chimpanzees (Knott, 1999), were also risk-seeking.

Interestingly, similar differences in risk preferences have been observed in human small-scale societies, possibly as an effect of cultural differences and environmental conditions (Kuznar, 2001; Henrich and McElreath, 2002) that deserves further investigation.

### Contextual and social factors affecting risk preferences in nonhuman primates

Several neurophysiological studies in nonhuman primates have employed risk preference tasks to understand whether single neurons track the subjective value rather than the objective value of a chosen option (McCoy and Platt, 2005; O’Neill and Schultz, 2010; So and Stuphorn, 2010; but see Yamada et al., 2013). In a first study, McCoy and Platt (2005) tested rhesus macaques (*Macaca mulatta*) in a visual gambling task and measured the activity of single neurons in the posterior cingulate cortex. Macaques were presented with choices between visual targets offering on average the same reward but differing in reward uncertainty. They had to choose whether directing their gaze to a safe target (offering a 150 ms access to fruit juice) or to a risky target (randomly offering either a shorter or longer than 150 ms access to juice, resulting on average in 150 ms access). Overall, monkeys strongly preferred the risky target and its selection increased with the degree of risk, regardless of the internal state of the subjects. Also neuronal activity increased with increasing variance in payoff of the risky option, mirroring the macaques’ risk proneness observed at the behavioral level. Interestingly, macaques continued to prefer the risky option even when the probability of receiving the larger outcome was reduced from 50 to 30% and thus its payoff was smaller than that of the safe option.

In the above study, rhesus macaques were consistently risk-seeking and the same pattern was observed also in subsequent studies carried out by the same Authors and in other neuro-physiological laboratories (Hayden et al., 2008b, 2010; Long et al., 2009; Watson et al., 2009; O’Neill and Schultz, 2010; So and Stuphorn, 2010; Heilbronner et al., 2011; but see Yamada et al., 2013). Interestingly, macaques’ choices are not explained by non-linear utility functions (as proposed by Lee, 2005) since they preferred an uncertain option, in which the delivery of the larger payoff was unpredictable, to an alternating option, in which the delivery of the larger payoff was predictably alternating across trials (Hayden et al., 2008a). Thus, borrowing the distinction between uncertainty and risk favored in the field of behavioral economics (Knight, 1921; Camerer and Weber, 1992; Tversky and Kahneman, 1992), macaques are not only risk prone, but also uncertainty-seeking.

However, not in all conditions do rhesus macaques exhibit a preference for risky options. In fact, when another macaque sample was tested in a risk preference task under different conditions, their behavior ranged from risk aversion to risk neutrality, but none of them was risk-seeking (Behar, 1961). Thus, although rhesus macaques’ ecology may suggest a general predisposition for risk proneness (Goldstein and Richard, 1989; Richard et al., 1989), Heilbronner and Hayden (2013) proposed that macaques’ risk preferences are driven by some features of the task design typically used in neurophysiological studies, such as (i) the small stakes involved in these experiments (typically 0.1–0.3 ml of juice); (ii) the large amount of trials (the same decision problem is typically presented hundreds or thousands of times to the same subject); and (iii) the short intertrial intervals (ITIs).

At least for the latter point, an experiment showed that this might be the case. Whereas in McCoy and Platt (2005), where macaques were risk-seeking, the average ITI was 3 s, in other nonhuman animal studies, where individuals were risk-averse (reviewed in Kacelnik and Bateson, 1996), the ITI was much longer (usually 30 s). Thus, Hayden and Platt (2007) presented rhesus macaques with a novel version of the visual gambling task in which the variance of the risky option was kept constant and the ITI varied from 1 s to 90 s. They found interestingly that, as the ITI increased, macaques’ preference for the risky option decreased and monkeys turned to risk neutrality at 90 s ITI. To explain this pattern, Hayden and Platt (2007) hypothesized that macaques interpreted the risky option as a certain reward available at a future time and, since the higher payoff may occur on the next trial, the subjective expected utility of the risky option depends on the length of the ITI. Interestingly, when humans were tested with a paradigm as similar as possible to that usually employed with macaques, they were more risk-seeking than in typical one-shot gambling experiments employing questionnaires (Hayden and Platt, 2009).

However, the above factors cannot explain the risk-seeking behavior observed in chimpanzees and orangutans (Heilbronner et al., 2008; Haun et al., 2011), where the stakes involved where comparatively high, the number of trials lower, and the ITIs longer than in the macaque studies. Although the results on chimpanzees appears to be very robust and have been replicated with larger samples (Rosati and Hare, 2012, 2013), it cannot generally be excluded that the different risk preferences obtained in the nonhuman primate studies reviewed so far were due to individual differences. In fact, in rhesus macaques, risk sensitivity appears to be partly determined by the serotonergic system: serotonin depletion increases risk proneness (Long et al., 2009), a finding consistent with recent rodent data (Koot et al., 2012). Similarly, the length polymorphisms of the serotonin transporter gene promotor (known as 5-HTTLPR, the serotonin-transporter-linked polymorphic region) is crucial as well (Watson et al., 2009), in relation to interspecific and intraspecific behavioral variability. Wendland and colleagues (2006) found, in macaque species, that the 5-HTTLPR was responsible for interspecific behavioral variability. In contrast, Chakraborty et al. (2010) proposed that this particular polymorphism had a role in intraspecific variability, which in turn may account for the greater ecological success of 5-HTTLPR polymorphic species. An example of its consequences in the wild is represented by the presumed selective emigration of rhesus macaques over the Himalyan Mountains into China in the early history of the species (Champoux et al., 1997; Heinz et al., 1998). According to Belsky et al. (2009), this particular polymorphism may confer an advantage when dealing with novel, possibly hostile environments. Relative to Indian-derived monkeys, Chinese-hybrid macaques with higher prevalence of the long repeat allele of the 5-HTTLPR show predispositions to aggressive and risk-taking behaviors, as well as lower levels of serotonin as indicated via its metabolite (Champoux et al., 1997; Heinz et al., 1998). Nonetheless, although feeding ecology and inter-individual differences are likely to influence risk preferences, the findings obtained in rhesus macaques underline the importance of carefully controlling all task and environmental parameters when comparing risk preferences among different species.

Finally, as observed in humans (Bault et al., 2008; Ermer et al., 2008; Hill and Buss, 2010), another important factor affecting nonhuman primates’ risk preferences seems to be the social context in which the individuals make decisions. To our knowledge, there is only one study evaluating this aspect in nonhuman primates (Rosati and Hare, 2012). Chimpanzees and bonobos were presented with choices between a safe option, yielding an intermediately preferred food item, and a risky option, yielding either a low-preferred or a high-preferred food item, in a competitive context and in a play context. In both contexts an experimenter interacted with the subject before the presentation of the decision-making task: in the competitive context, the experimenter first offered the subject a food item and then, when the subject attempted to take it, immediately pulled it out of the subject’s reach; in the play context, the experimenter tickled or chased the subject. Apes’ behavior in each condition was compared with a neutral context, in which the experimenter was present but not interacting with the subject. All subjects chose the risky option more in the competitive than in the neutral context, whereas the play context did not increase risk proneness. Probably, an eco-ethological explanation is very likely given that feeding competition and consequent loss of resources is a potential problem for all group-living species. In this frame it can be proposed that, in the competitive context, the salience and attractiveness of the larger option would be increased notwithstanding its uncertainty.

### The evolutionary origins of biases in decisions under risk

When making choices between risky options, humans show the so-called “reflection effect”, i.e., the tendency to evaluate gambles in relation to an arbitrary reference point. The same individual can decide differently, being risk-seeking when some options are framed as losses and risk-averse when the same, identical options are framed as gains (Kahneman and Tversky, 1979; Tversky and Kahneman, 1981).

Nonhuman animals apparently share with humans the reflection effect and other behavioral biases (e.g., Waite, 2001; Marsh and Kacelnik, 2002; Shafir et al., 2002). This can be either because of an early emergence of economic biases during evolution, or because of convergent evolution. Only the study of nonhuman primates, our closest relatives, can allow to disentangle the topic and select one between these two hypotheses. To this aim, in recent years a series of studies investigated decision-making under risk in capuchin monkeys (*Sapajus* spp., formerly *Cebus apella*^2^) that, despite 35 million year of independent evolution, show many striking analogies with humans in terms of encephalization index, ontogeny, lifespan, and various cognitive traits (Fragaszy et al., 2004).

In a first study (Chen et al., 2006), capuchins were tested in a token exchange task, in which they were provided with a starting budget of 12 tokens that could be exchanged with one of two experimenters, as they preferred. Preliminary experiments demonstrated that capuchins can behave rationally in this framework: when the two experimenters provided the same amount of two equally preferred different food types, capuchins exchanged a similar amount of tokens with each of them; however, when one experimenter doubled the amount of food provided in exchange for one token or showed two food items and delivered either one or two pieces with the same probability, capuchins reliably shifted their preference towards her, showing that they were able to maximize their payoff. In the main experiment, capuchins were presented with choices between experimenters providing a risky “trade” of either one or two food items with equal probability, but the amount of food initially displayed to the subject was different: one experimenter showed one food item and added a “gain” of one additional food item in half of the trials, whereas the other experimenter showed two food items and subtracted a “loss” of one food item in half of the trials. Although the two experimenters provided on average the same payoff, capuchins preferred to exchange their tokens with the first experimenter, although—according to a rational perspective—they should have been indifferent between the two options. These results demonstrate that, as in humans, they chose on the basis of an arbitrary reference point (namely, the initial food amount shown by the two experimenters), therefore preferring the experimenter which was framing the “trade” as a gain.

In a subsequent study (Lakshminarayanan et al., 2011), capuchins were tested with a similar paradigm, presenting them choices between a risky option and a safe option yielding the same average payoff (of two food items) but in two conditions: (i) *Losses*: both experimenters initially displayed three food items, but the first experimenter always delivered two food items, whereas the second experimenter delivered either one or three food items with equal probability; and (ii) *Gains*: both experimenters initially displayed one food item, but the first experimenter always delivered two food items, whereas the second experimenter delivered either one or three food items with equal probability. Overall, capuchins showed a clear-cut evidence of the “reflection effect” since they were risk-seeking when options were framed as losses, and risk averse (although to a lesser extent) when options were framed as gains. Again, decisions appear to be made by subjects relative to their initial reference point.

In sum, the above findings suggest that humans and capuchin monkeys share the reflection effect, as is reported with other behavioral biases (Chen et al., 2006; Lakshminarayanan et al., 2008). However, a very recent “up-linkage” replication of Lakshminarayanan et al. (2011), in which adult humans were tested with exactly the same procedure employed with capuchin monkeys, failed to find a reflection effect (Silberberg et al., 2013). Nonetheless, it should be noted that such a replication may have had a low ecological validity for cognitively sophisticated adult humans, especially because of the repeated interactions with the experimenters, which the participants may have found boring or embarrassing. Future studies should investigate biases in decisions under risk in closely-related non-human primate species with different ecologies (Clutton-Brock and Harvey, 1979; Rosati and Stevens, 2009; Rosati and Hare, 2012) in order to understand whether these behavioral patterns are maladaptive, suboptimal, or instead “ecologically rational” (Todd and Gigerenzer, 2000).

## Risk attitudes, environmental uncertainty and addictive behavior: perspectives from computational neuroscience and evolutionary robotics

Computational models are a new way of doing science which can be very useful for theorizing about extremely complex systems like vertebrate organisms and their brains. The usefulness of computational models comes largely from two factors: (i) they express hypotheses in a formal, precise, and unambiguous way, so that from those hypotheses a number of detailed predictions can be unequivocally derived which can then be tested through empirical experimentation; (ii) they allow for a degree of direct manipulation on all relevant variables which is unparallelled by naturalistic methods.

The vast majority of computational models deal with the normal functioning of the brain and normal cognitive phenomena, but since the 1990s a number of models have been proposed that address psychiatric and neurological disorders, and recently these models have been raising increasing interest, so that several scholars started to discuss the prospects, challenges, and limitations of *computational psychiatry* (Maia and Frank, 2011; Montague et al., 2012; Huys, 2013). There are many ways in which computational models may help research on decision-making in general and pathological gambling more in particular. Here, we will focus on three different kinds of models: (1) normative (algorithmic) models; (2) neural models; and (3) evolutionary robotics models.

### Normative (algorithmic) models

A first class of relevant models is what we can call “normative” or “algorithmic” models. These models derive from the computational reinforcement learning literature (Sutton and Barto, 1998) and are normative because they are based on machine learning algorithms, which prescribe how an agent *should* behave in order to maximize its payoff with future rewards. They became famous in the mid 1990s when it was discovered that the dynamics of dopamine, which is highly involved in motivation and learning (Wise, 2004; Schultz, 2006; Berridge, 2007), as well as in drug addiction, could be modeled by the *reward prediction error* signal postulated in Temporal Difference (TD) reinforcement learning (Barto, 1995; Schultz et al., 1997). The reward prediction error of TD learning is a signal that quantifies “surprise”, that is, the difference between expected and actual rewards, and it is used in reinforcement learning models as the learning signal that drives action learning. In a nutshell, the theory holds that an agent continually evaluates the current states (situations) with respect to the reward that it expects to achieve in those states. If it gets more reward than expected, then a prediction error signal is generated that is used to update both its prediction and its action policy, that is the way the animal selects its actions. The idea is that the probability to select an action again, in a given context, is increased if that action leads to more rewards than expected and is decreased if it leads to less reward than expected. Dopamine behaves just as the reward prediction error: its release is triggered by unexpected rewards or unexpected stimuli that predict reward but it is not released when the reward is perfectly predictable and it is inhibited (a deep in dopamine levels occurs) when an expected reward is omitted. This has led to conclude with the hypothesis that dopamine plays the same function of the reward prediction error, within phenomena of reinforcement. Phasic dopamine release would have the role of making the agent learn (1) the value (“saliency”) of the stimuli and (2) which are the actions (“strategies”) to be deployed in each circumstance in order to maximize future rewards. In mammals, these two roles are attributed to mesolimbic vs. nigrostriatal dopamine pathways, respectively. This theory has guided an enormous amount of empirical research and has received so much empirical support that it is now an important tenet of contemporary neuroscience, and it has become one of the most successful examples of using computational models in the behavioral and brain sciences (e.g., Montague et al., 2004; Ungless, 2004; Wise, 2004; Sugrue et al., 2005; Graybiel, 2008; Glimcher, 2011).

What is most interesting for our purposes is that the reward prediction error hypothesis for dopamine has not only been used to predict and explain behavioral and brain dynamics in normal conditions, but also to explain pathological phenomena. In particular, normative algorithmic models have been used to interpret brain imaging data related to various mental pathologies like schizophrenia and depression-related anhedonia (Smith et al., 2007; Kumar et al., 2008; Murray et al., 2008; Huys et al., 2013).

Moreover, a seminal work by David Redish (2004) used a TD model to explain drug addiction. In particular, the model explained addiction as the consequence of the pharmacological effect that certain drugs of abuse, like amphetamines, cocaine or nicotine, may have on forebrain dopamine circuits. Indeed, these drugs are known to increase dopamine levels upon acute administration. According to Redish’s model, the addictive effect of these drugs is associated to specific consequences, due to the dopamine elevation produced by the drug. With natural rewards, a phasic release of dopamine is present only when the reward is not predicted, unexpected. In this perspective, the normal process of reinforcement, produced by any reward, can be cancelled out by accurate predictions. On the contrary, the model postulates that drugs of abuse generate also a pharmacologically-induced dopamine release, a term that cannot be compensated by predictions. Since, in this way, the dopamine prediction error never disappears, as if drug-related pleasure is always “unexpected”, the subjective values of the drug related internal states will keep on increasing indefinitely, and the actions that lead to the drug consumption keep on being reinforced, hence becoming a strong habit and thus ultimately resulting in the development of addiction. This model explains several aspects of addiction including, for example, the fact that both drugs and natural rewards are sensitive to effort-related cost, but the reward provided by drugs is much less sensitive than that given by natural rewards. However, one of the key predictions of the theory has been falsified by subsequent research. In particular, the theory predicted that drugs should prevent blocking, i.e., the phenomenon for which a stimulus that predicts a reward, if paired with a new stimulus before presenting the reward, prevents the second stimulus to be conditioned as it stops the learning-inducing dopamine prediction error from occurring. If a drug always produced a dopamine prediction error, as postulated by Redish’s model, then the conditioning of the second stimulus should occur, but it does not (Panlilio et al., 2007).

Building on this computational interpretation of drug addiction, Redish et al. (2007) proposed a model that provides a possible explanation of pathological gambling. This model adds to the basic TD prediction error model, which learns the values of states and actions, a second “situation recognition” system that learns to categorize the states. In particular, this system learns to categorize as different states all those situations in which, after having received high rewards, those rewards are not present anymore. Noteworthy, this addition was done to accommodate in the TD framework basic reinforcement learning phenomena related to the extinction of behaviors and their renewal. However, it provides also an explanation of gambling. Indeed, many pathological gamblers became addict after having experienced an unlikely sequence of wins or a single very high win (Custer, 1984; but see Kassinove and Schare, 2001, for empirically founded doubts on the strength of this big win effect). The model assumes that, when the gambler experiences such a huge success (or the feeling to have almost succeeded, the so called “near miss” effect; Kassinove and Schare, 2001), he forms a very strong and unrealistic expectation that he can win again (or finally; on the similarity in neural processing of wins and near misses, see Chase and Clark, 2010; Winstanley et al., 2011). When the gambler starts to loose, instead of unlearning and cancelling this (false) expectation, by negative reinforcement, his situation recognition system starts to create new “associative” states, namely looking for cues that are supposed to distinguish the winning situation against the loosing ones. Hence, according to this model, pathological gambling results from a misclassification of the situation, with the irrational belief that there are contingencies in which the gambler can win as different from those where he looses. This explanation can account also for two related phenomena: (1) the “hindsight bias” effect, where gamblers analyze their losses and (*post-hoc*) identify which are the cues that differed from the situation when they won, as well as (2) the “illusion of control” phenomenon, in which they believe that they can control an otherwise random situation by identifying and following the right cues that, in their mind, distinguish winning from losing situations (Custer, 1984; Wagenaar, 1988). The most common superstitions of pathological gamblers are thus accounted for.

One limitation of this model is that it tries to explain pathological gambling as a unitary phenomenon with a unique cause, while it is likely that there might be several different causes that underlie this complex behavior, both in the same individual and across different individuals. For example, many pathological gamblers keep on gambling even if they report knowing that they will loose, something that is in contrast with the model (but see the results on cue-induced secondary rewards in rodents and their potential implications for human gambling, discussed in Section Assessment of Gambling Proneness: Clinical and Preclinical Approaches). However, the most important limit of this kind of normative, algorithmic models is that they provide abstract explanations on what computations may go awry in pathological conditions, but they do not explain which are the actual brain mechanisms that may underlie these phenomena: hence the range of phenomena that they can account for and predict is limited. In order to investigate the details of the brain processes that are the basis of the phenomena of study, we need models that simulate those details. This is the province of neural models.

### Neural models

Neural models explain a cognitive phenomenon by simulating (with a variable degree of abstraction) neurons and their connections, and making the simulated neural network reproduce the phenomenon. The first models of this kind were called “connectionist” models (McClelland and Rumelhart, 1989): they included very simple neural networks, which were supposed to perform computations in a brain-like manner, but whose structure was not meant to replicate the structure of real brains. More recently, much more biologically realistic models have been developed in computational neuroscience. In these models, different groups of nodes are meant to represent neurons belonging to different parts of the brain, and the connections between the different groups correspond to the connections between those brain areas. The architecture and functioning of the model are thus based on the anatomy and physiology of the same brain areas that are known to be relevant for the phenomenon under study. If the model is able to reproduce the phenomenon, this would give us a detailed explanation on what brain mechanisms may be responsible for it. The plausibility of such an explanation rests on two foundations: (i) how many anatomical and physiological constrains are considered, and how much they are respected; and (ii) how many different phenomena the model is able to account for. Furthermore, the model can be used to derive a number of predictions that can then be tested in humans as well as animal models, through further empirical experiments.

To the best of our knowledge, no neural models have been developed so far to explain pathological gambling, although there is evidence of a role of midbrain dopamine in the coding of reward uncertainty (Fiorillo et al., 2003), thus suggesting an influence of the dopaminergic system on risk-taking behavior. On the other hand, several models, both connectionist (e.g., Cohen and Servan-Schreiber, 1992; Cohen et al., 1996; Braver et al., 1999) and biologically detailed ones (Frank et al., 2004, 2007a,b,c; Gutkin et al., 2006; Waltz et al., 2007; Rolls et al., 2008; Ahmed et al., 2009; Maia and Frank, 2011), have been developed to describe neurological and psychiatric pathologies, including schizophrenia, Parkinson, Tourette’s syndrome, ADHD, and drug addiction. Briefly reviewing these existing models can provide useful suggestions on how to apply the same methods to the investigation of pathological gambling.

Most of these models deal with the dopaminergic system and its interactions with the basal-ganglia-thalamo-cortical circuits that implement action selection. A notable example is the work of Frank and colleagues on modeling several aspects of Parkinson disease (e.g., Frank et al., 2004, 2007a; Moustafa et al., 2008). Parkinson disease is known to depend on the degeneration of nigro-striatal dopamine cells. This work is based on a detailed model of the basal ganglia-thalamo-cortical circuit that is assumed to implement action selection and reinforcement learning (e.g., Frank et al., 2001). The main idea behind the model is that two sub-systems, a Go and a no-Go system, are present in the basal ganglia, which together implement action selection. In particular, neurons in the basal ganglia are supposed to allow the release of actions in the cortex by selectively disinhibiting a certain action (through the Go system) while inhibiting the others (through the no-Go system). Furthermore, a third structure of the basal ganglia (the subthalamic nucleus) is supposed to dynamically exert a global inhibitory role and to modulate the threshold at which actions are selected depending on the level of cortical conflict. Importantly, neurons belonging to the different systems have different dopamine receptors distributions, with Go neurons having receptors which make dopamine excite the neuron and no-Go neurons that have receptors which make dopamine inhibit the neuron. Through such a model, Frank and colleagues have been able to reproduce and explain a number of detailed behavioral and neural data, and to predict new data that have been empirically verified, such as the effects of dopaminergic medication and of deep brain stimulation of the subthalamic nucleus (a procedure that is known to improve motor symptoms) on different cognitive tasks in Parkinson patients (Frank et al., 2007a), and why medication can lead those patients to develop pathological gambling (Dodd et al., 2005).

In order to explain other facets of this complex behavior and its neural basis, many more details should be added to these models. For example, pathological gambling is known to be associated with dysfunction not only of dopamine, but also of other neuromodulators like serotonin (e.g., Nordin and Eklundh, 1999) and noradrenaline (e.g., Meyer et al., 2004). For this reason, the role of these two neuromodulators should be modeled in future research, possibly by incorporating findings from other computational models that deal with the interactions between these neuro-modulators and dopamine (e.g., Daw et al., 2002). Furthermore, beyond the anomalies in the basal-ganglia and in associated fronto-cortical areas, recent evidence suggests that also deficits in amygdala functioning may be responsible for gambling behavior by significantly reducing loss aversion (De Martino et al., 2010). For this reason, modeling pathological gambling may require modeling the interactions between the amygdala and the basal-ganglia, as done in recent neuro-robotic models of the role of amygdala in conditioning (Mannella et al., 2007, 2008, 2010; Mirolli et al., 2010).

Finally, also factors related to *intrinsic motivations* (i.e., motivations related to novelty, surprise, and competence acquisition: Ryan and Deci, 2000; Baldassarre and Mirolli, 2013) may play a role in pathological gambling. For example, Parkinson patients that develop pathological gambling are distinguished from those that do not in tests that measure impulsivity and novelty seeking (Voon et al., 2007). Recent computational models assume that intrinsic motivations work by hijacking the neural brain systems that underlie also extrinsic motivations, and in particular the dopaminergic system and the action selection system in the basal-ganglia (e.g., Kakade and Dayan, 2002; Mirolli et al., 2013). Some of these models are detailed neural models very similar to the ones discussed above on dopamine in Parkinson, including basal-ganglia-thalamo-cortical circuits, the dopaminergic system, and other relevant areas (e.g., Baldassarre et al., 2013; Fiore et al., 2014). Merging the two kinds of models may be a promising way to further understand the brain mechanisms underlying pathological gambling.

### Evolutionary robotics models

Evolutionary robotics provide a valuable platform to test evolutionary hypotheses on the ecological pressures behind the emergence of specific behaviors and traits. Such hypotheses, like those already discussed in Sections Rodents as an Animal Model of Gambling Behavior and Risky Choices in Nonhuman Primates: Implications for Human Pathological Gambling with respect to risk attitudes, are often plausible, but also hard to verify directly. They rely on key assumptions about the environment in which the evolution of a given species occurred, and yet it is typically hard to observe with precision the effects of a given ecological variable (e.g., dangers of predation) on the behavior under study (e.g., risk proneness/aversion). Moreover, these assumptions refer to ancestral environments, not present-day ecologies: while there are methods to acquire data on living conditions in ancestral times (e.g., through paleobiology and primate archeology; Haslam et al., 2009), they are bound to deliver incomplete information at best, in spite of substantial research efforts. Recent work has demonstrated the viability and fruitfulness of computational methods, e.g., experimental evolutionary robotics: the basic idea is to let populations of simulated robots evolve under specific ecological pressures, and then observe their behavior with the aim of drawing implications for the understanding of processes in natural organisms faced by similar, uncertainty-based tasks (Da Rold et al., 2011; Saglimbeni and Parisi, 2011). This approach allows to observe how several forms of risk introduced in the evolutionary environment affect choice behavior, both in ecology and in experimental settings.

Moreover, robots are controlled by simple neural networks, whose evolution and effects on behavior can be studied with extreme precision and flexibility: not only recording their activity during behavior, but also “lesioning” a well-adapted neural network and observing the impact on risk-related choices, hence drawing new insights into pathological gambling. These are all key advantages of computational evolutionary models, as opposed to purely mathematical and game-theoretical approaches, for putting forward hypotheses regarding the evolution of certain aspects of risk attitudes in uncertain environments (e.g., McNamara et al., 2013). While mathematical and theoretical models certainly provide valuable contributions to breach the gap between laboratory studies and ecological observations, they lack the opportunities for direct manipulation and experimental observation granted instead by robotics platforms, be they purely simulated or physically implemented.

To the best of our knowledge, no evolutionary (computational) model of pathological gambling have yet been proposed. However, there are several interesting simulations on how risk attitudes in general might have evolved: some of these works have already important implications for our understanding of gambling behavior, and points towards promising research directions. For instance, Niv et al. (2002) used evolutionary computation techniques to evolve near-optimal neuronal learning rules in a simple neural network model of reinforcement learning in bumblebees foraging for nectar. This resulted in a replication of two well-documented choice strategies in these animals: risk aversion and probability matching. Moreover, risk aversion evolved even in a completely risk-less environment. These results suggest that risk-aversion may be a direct consequence of near-optimal reinforcement learning, with no need to assume further evolutionary constraints, such as the existence of a nonlinear subjective utility function for rewards. Their results were also demonstrated in real-world situations, using experiments in a Kephera wheeled robot, and they dovetail nicely with the evidence on the role of the reward prediction error in determining various choice behaviors (see Section Normative (Algorithmic) Models).

Other models do not explicitly focus on any particular species, but rather try to address general issues pertaining the evolution of risk attitudes. Arbilly et al. (2011) used agent-based evolutionary simulations to investigate an important connection between environmental features, risk-aversion, and the evolution of social learning. They started from the observation that, in environments with significant risks associated to higher value rewards (e.g., an ecology in which the most valuable food is rare and difficult to obtain), the possibility of acquiring such rewards is most likely to require a certain number of failed attempts, before success is achieved. In these circumstances, risk-aversion would lead to neglect such rewards, even if doing so may be sub-optimal in the long run (Real, 1991). However, Arbilly and colleagues noted that this situation also create an important (and often overlooked) evolutionary advantage to social learning over individual learning, since social learners can by-pass the problem of risk aversion by learning where to forage from individuals that have already found food. The results of their evolutionary simulations, which combined a producer–scrounger game with explicit individual and social learning rules for associating different food patch types with experienced reward, confirmed the key role of social learning in similar situations, as an antidote to the adverse effects of risk-aversion in this type of environment. Incidentally, this also provides an explanation to why many species, humans included, continue to rely heavily on social learning even when it produces disastrous effects, e.g., in escape panic scenarios (Helbing et al., 2000). And it also illustrates how this reliance on social learning can be used to produce “contagious gambling”: this is precisely what happens when con-artists and casinos employ confederates who (falsely) win huge sums, in order to lure unsuspecting potential gamblers into the game.

While the number of computational evolutionary models of risk attitudes is still too limited to permit any universal conclusions on the evolution of this complex suite of behaviors, some important methodological implications stand out, and are worth noticing. This methodology has in fact both advantages and limitations, but what matters is that they tend to be complementary to those exhibited by naturalistic methods. Thus, integrating evolutionary simulations with naturalistic studies has the potential for huge scientific payoffs. With respect to experimental evolutionary robotics (Da Rold et al., 2011; Saglimbeni and Parisi, 2011), advantages of this method include the following ones. First, full observability means that robots’ behavior can be observed in extreme detail both “in the wild” (i.e., in the ecological setting where robots evolve), and “in the lab” (i.e., under specific test conditions). Second, there is full control, meaning that all variables can be easily and precisely manipulated, regarding both ecology and test conditions, including the possibility of “counterfactual experiments” (that is, studying how ecological pressures for which no natural correlate is known might affect behavior). Third, there is neurocomputational transparency, in that also the internal dynamics of the robots’ control system (e.g., a neural network) are precisely measured (which is not entirely the case for natural, alive organisms). Fourth, individual differences emerge, since robots differ in how they cope with their ecology and in their level of proficiency (also, opening the way to the study of artificial pathologies). Interestingly, non-deterministic responses are present, since evolutionary robots are typically responding in a non-deterministic way, with respect to external stimuli, facilitating comparison with natural, alive organisms (who also do not react always in the same way to identical inputs from the environment). Finally, a potential exists for embodied implementation, since simulated robots are based on simulators of real physical platforms, thus allowing easy implementation in real-world scenarios.

In contrast, the method is mostly vulnerable to the following problems and limitations. First, abstraction, since both the ecology and the artificial laboratory are much simpler than most natural counterparts (and the same is true for the structure of the robot’s body and its control system). Second, there is much arbitrariness, since a huge variety of parameters needs to be set by the experimenter, concerning both the ecology, the robot’s structure, and the test conditions (and these are likely to have some impact on the resulting behavior). Last, there is need to start small; however, given the number of variables directly controlled by the experimenter and the amount of data obtained, a scalar approach is unavoidable (to understand the results). As mentioned, however, most of these drawbacks can be easily overcome, by allying computational evolutionary models with naturalistic studies (see Sections Risky Choices in Nonhuman Primates: Implications for Human Pathological Gambling and Risk Attitudes, Environmental Uncertainty and Addictive Behavior: Perspectives from Computational Neuroscience and Evolutionary Robotics).

## Conclusions

In this review, we first discussed how the development of refined operant protocols, to reproduce and to evaluate the gambling proneness phenotype in animal models, is fundamental to increase our understanding of the neurobiological determinants underlying the etiology of pathological gambling and/or to develop new treatment strategies. Then, we surveyed the role of comparative studies on choice behavior in other species, in particular in nonhuman primates, for informing us on the evolutionary origins and cognitive underpinnings of human attitudes towards risk and uncertainty. Finally, we summarized various ways in which computational models can be of assistance in the study of gambling behaviors: while results in this area are still preliminary, we were able to point out several substantial indications originated from combining naturalistic observations and artificial modeling.

Reviewing such diverse studies together is meant to impact on the methodology of future gambling research: while looking at each of these three rich areas of research in isolation is certainly useful, the potential emerging benefits are only compounded by integrating all these methods together. What one learns from an animal model (about the neurobiological underpinnings of pathological gambling) should immediately be verified via computational techniques, and the further predictions generated by that computational model should be tested empirically in natural, alive organisms. Similarly, any evolutionary hypothesis on what adaptive pressures shaped risk attitudes, and generated (possibly as a by-product) gambling behavior, should be verified via computational evolutionary models, which in turn should be informed by naturalistic data coming from ethological studies. Only by bringing to the table both human and nonhuman gamblers, we shall understand what makes us so vulnerable to such a self-destructive behavioral pattern.

## Conflict of interest statement

The authors declare that the research was conducted in the absence of any commercial or financial relationships that could be construed as a potential conflict of interest.

## Footnotes

^1^Another common way of distinguishing between risk and uncertainty is in terms of how measurable the odds are: Knight (1921) proposed to consider as “risky” those choices were the odds are measurable and known to the subject, whereas the term “uncertainty” should be reserved for probabilistic outcomes with unknown odds. While this distinction has become canonical in behavioral economics (e.g., Camerer and Weber, 1992; Tversky and Kahneman, 1992), its application to animal studies is highly problematic, due to obvious difficulties in establishing how much the odds are known (that is, precisely understood and quantitatively assessed) by experimental subjects.

^2^Recent molecular analysis has revealed that capuchin monkeys, formerly identified as the single genus *Cebus*, are two genera, with the robust (tufted) forms (including libidinosus, xanthosternos, apella and several other species) now recognized as the genus *Sapajus*, and the gracile forms retained as the genus *Cebus* (Lynch Alfaro et al., 2012). The nomenclature for *Sapajus* is registered with ZooBank (urn:lsid:zoobank.org:act:3AAFD645-6B09-4C88-B243-652316B55918). Animals identified as *Cebus apella* in laboratory colonies outside of South America may be any combination of the several species (e.g., *C. apella*, *C. libidinosus*, *C. nigritus*) recognized as separate species since 2001 (Groves, 2001; Fragaszy et al., 2004), but previously considered *C. apella*.
